# Comparing Different Methods for the Diagnosis of Liver Steatosis: What Are the Best Diagnostic Tools?

**DOI:** 10.3390/diagnostics14202292

**Published:** 2024-10-16

**Authors:** Sophie Chopinet, Olivier Lopez, Sophie Brustlein, Antoine Uzel, Anais Moyon, Isabelle Varlet, Laure Balasse, Frank Kober, Mickaël Bobot, Monique Bernard, Aurélie Haffner, Michaël Sdika, Bruno Montcel, Benjamin Guillet, Vincent Vidal, Emilie Grégoire, Jean Hardwigsen, Pauline Brige

**Affiliations:** 1 Department of Digestive Surgery and Liver Transplantation, Hôpital la Timone, AP-HM, 13005 Marseille, France; jean.hardwigsen@ap-hm.fr; 2 Aix Marseille Université, LIIE, 13007 Marseille, France; olivier-lopez@live.fr (O.L.); vincent.vidal@univ-amu.fr (V.V.); eg.chirurgie@gmail.com (E.G.); pauline.brige@univ-amu.fr (P.B.); 3 Aix Marseille Université, CERIMED, 13007 Marseille, France; sophie.brustlein@univ-amu.fr (S.B.); laure.balasse@univ-amu.fr (L.B.); mickael.bobot@gmail.com (M.B.); benjamin.guillet@univ-amu.fr (B.G.); 4 Department of Digestive and Oncologic Radiology, Hôpital l’Archet 2, 06202 Nice, France; 5 INSA-Lyon, Université Lyon 1, UJM-Saint Etienne, CNRS, Inserm, CREATIS UMR5220, U1294, 69616 Lyon, France; antoine.uzel@creatis.insa-lyon.fr (A.U.); michael.sdika@creatis.insa-lyon.fr (M.S.); bruno.montcel@creatis.insa-lyon.fr (B.M.); 6 C2VN, INSERM 1263 INRAE 1260, Aix-Marseille Université, 13007 Marseille, France; anais.moyon@univ-amu.fr; 7 Department of Radiopharmacy, Hôpital la Timone, AP-HM, 13007 Marseille, France; 8 Aix-Marseille Université, CNRS, CRMBM, 13007 Marseille, France; isabelle.varlet@univ-amu.fr (I.V.); frank.kober@univ-amu.fr (F.K.); monique.bernard@univ-amu.fr (M.B.); 9 Center of Nephrology and Kidney Transplantation, Hôpital de la Conception, AP-HM, 13005 Marseille, France; 10 Department of Anatomopathology, Hôpital la Timone, AP-HM, 13007 Marseille, France; aurelie.haffner@ap-hm.fr; 11 Aix-Marseille Université, 27 Boulevard Jean Moulin, 13385 Marseille, France

**Keywords:** liver steatosis, MCD diet, CARS microscopy, MR spectroscopy, ^99m^Tc MIBI, near-infrared spectroscopy

## Abstract

Background: Due to the ongoing organ shortage, marginal grafts with steatosis are more frequently used in liver transplantation, leading to higher occurrences of graft dysfunction. A histological analysis is the gold standard for the quantification of liver steatosis (LS), but has its drawbacks: it is an invasive method that varies from one pathologist to another and is not available in every hospital at the time of organ procurement. This study aimed to compare non-invasive diagnostic tools to a histological analysis for the quantification of liver steatosis. Methods: Male C57BL6J mice were fed with a methioninecholine-deficient (MCD) diet for 14 days or 28 days to induce LS, and were compared to a control group of animals fed with a normal diet. The following non-invasive techniques were performed and compared to the histological quantification of liver steatosis: magnetic resonance spectroscopy (MRS), CARS microscopy, ^99m^Tc MIBI SPECT imaging, and a new near-infrared spectrometer (NIR-SG1). Results: After 28 days on the MCD diet, an evaluation of LS showed ≥30% macrovesicular steatosis. High correlations were found between the NIR-SG1 and the blinded pathologist analysis (R^2^ = 0.945) (*p* = 0.001), and between the CARS microscopy (R^2^ = 0.801 (*p* < 0.001); MRS, R^2^ = 0.898 (*p* < 0.001)) and the blinded pathologist analysis. The ROC curve analysis showed that the area under the curve (AUC) was 1 for both the NIR-SG1 and MRS (*p* = 0.021 and *p* < 0.001, respectively), while the AUC = 0.910 for the Oil Red O stain (*p* < 0.001) and the AUC = 0.865 for the CARS microscopy (*p* < 0.001). The AUC for the ^99m^Tc MIBI SPECT was 0.640 (*p* = 0.013), and this was a less discriminating technique for LS quantification. Conclusions: The best-performing non-invasive methods for LS quantification are MRS, CARS microscopy, and the NIR-SG1. The NIR-SG1 is particularly appropriate for clinical practice and needs to be validated by clinical studies on liver grafts.

## 1. Introduction

Liver steatosis (LS), also known as non-alcoholic fatty liver disease (NAFLD), is a metabolic dysfunction that results from an accumulation of triglyceride-containing lipid droplets in hepatocytes. Steatosis is considered pathologic when fat accumulation in the liver exceeds 5%. The evolution of NAFLD leads to non-alcoholic steatohepatitis (NASH), cirrhosis, or liver failure [1]. Liver transplantation (LT) is the reference treatment for end-stage liver disease. Due to the ongoing organ shortage, marginal liver grafts with steatosis are more frequently used in liver transplantation, leading to a higher allograft dysfunction rate and poor outcomes [2]. Recently, after an international panel of experts led a consensus-driven process to develop a more appropriate term for the disease, the term NAFLD was replaced by the term metabolic dysfunction-associated steatotic liver disease (MASLD) [3]. In fatty liver disease (FLD), two types of liver steatosis have been identified: microvesicular (mV) and macrovesicular (MV) steatosis. These are differentiated by the size of the lipid droplets and the deviation of the hepatocyte nuclei. Only MV liver steatosis has an impact on liver function [4]. MV is graded into three categories according to the proportion of hepatocytes containing liver droplets: mild at ≤30%, moderate at 30–60%, and severe at ≥60%. Moderate-to-severe liver steatosis in a liver graft leads to higher liver dysfunction and is associated with poorer recipient survival after liver transplantation [5].

The gold standard for the quantitative assessment of MV liver steatosis is a histological analysis, which can vary depending on the pathologist, and is not available in every hospital at the time of donor procurement. There is a lack of reliable diagnostic tools with which to identify liver steatosis and to quantify MV liver steatosis with non-invasive techniques. Various techniques have been assessed and proposed as alternatives to a liver biopsy, but none are currently validated. These proposed techniques include ultrasound (US), CT scan, magnetic resonance spectroscopy [6], fibroscan, CAP (controlled attenuation parameter) [7,8], metabolic (99m)Tc-MIBI SPECT imaging [9], Raman spectroscopy or coherent anti-Stokes Raman scattering (CARS) microscopy, and near-infrared spectroscopy (NIRS). MRIs have shown the best performance among the non-invasive imaging methods for LS quantification, but they are available less frequently, especially in cases of organ procurement [10]. (99m)Tc-MIBI is a lipophilic cation designed for myocardial perfusion scintigraphy in the diagnosis of ischemic heart disease, and its retention reflects mitochondrial function [11]. Raman spectroscopy (RS) or coherent anti-Stokes Raman scattering (CARS) microscopy is a method that detects the vibration state of the C–H bonds in triglycerides. CARS microscopy can be used to quantify LS and for the identification of micro- and macrovesicular steatosis by detecting lipid droplets and quantifying their number and size [12,13].

Near-infrared spectroscopy (NIRS) is a method of diffuse reflectance spectroscopy (800–2000 nm) that has emerged as a promising alternative technique for the assessment of LS [14]. Moreover, NIRS has the potential to provide quantitative fat measurements, enhancing the precision of steatosis assessment compared to conventional imaging techniques. NIRS is a reliable technique with which to assess LS [15], and transplantation teams have developed miniaturized NIRSs with promising results for the quantification of LS [16].

The aim of this study was to compare different diagnostic tools (^(99m)^Tc-MIBI, CARS microscopy, MRS, ORO, and NIRS) to an anatomopathological quantification of liver steatosis in a murine model.

## 2. Materials and Methods

### 2.1. Animals and Experimental Design

All the procedures involving animals were approved by the Institution’s Animal Care and Use Committee (CEEA71, Aix-Marseille Université) under reference #16164 and CEEA14#24447, and were conducted according to the 2010/63/EU European Union Directive and the ARRIVE guidelines 2.0 [17]. The mice were housed in enriched cages and placed in a temperature- and hygrometry-controlled room with daily monitoring. They were provided with water and a commercial diet ad libitum.

Seven-week-old pathogen-free male C57BL/6 mice were purchased from Janvier Labs, France. After a 7-day acclimatization period, the mice were arbitrarily divided into a control group, which were fed with a standard diet (SAFE^®^ D03), and an interest group, which were subject to an MCD regime (SAFE^®^ MCD 0174).

**Figure 1:**
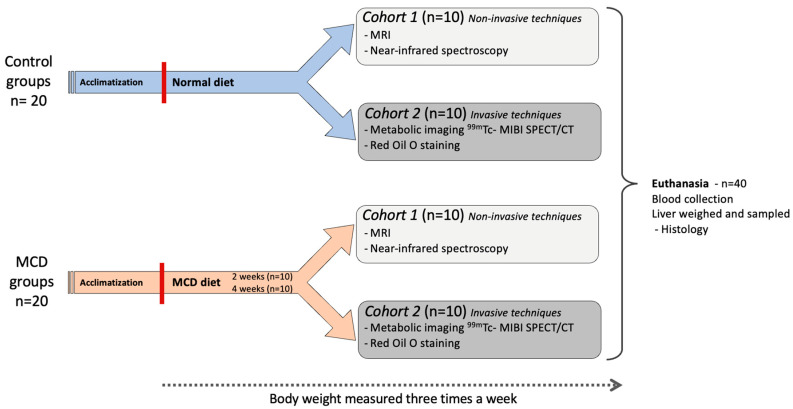
Experimental study design.

A total of 40 animals were included in this study. Two cohorts of 20 animals each were used; these were divided into a control group (*n* = 10), an MCD-14d group (*n* = 5) administered the diet for 14 days, and an MCD-28d group (*n* = 5) administered the diet for 28 days. One cohort was used to evaluate the following invasive methods: ^99.m^Tc-MIBI SPECT/CT and Oil Red O staining (ORO). The other cohort (*n* = 20) was used to evaluate the following non-invasive methods: MRS, CARS microscopy, and NIRS (Figure 1). At the end of the experiment, the mice were euthanized through cardiac blood collection; following this, the liver tissue was weighed and sampled. For all imaging sessions and prior to the euthanasia procedure, the mice were anesthetized by means of an intraperitoneal injection of ketamine (100 mg/kg) and xylazine (10 mg/kg).

### 2.2. Clinical and Biological Evaluation

During the experiment, the body weight (BW) was measured for all the mice, and the weight loss of the animals was assessed three times per week. The liver weight (LW) was determined at the time of euthanasia, as was the liver-to-body weight ratio (LBW ratio).

#### Blood Sampling and Analysis

Following the administration of the standard diet (control) or the MCD diet for 2 or 4 weeks, blood samples were collected from the vena cava after anesthesia and separated by means of centrifugation (3000× *g*, 4 °C, 15 min) to collect the serum. Alanine aminotransferase (ALT) activity (*N* < 50 UI/L) was analyzed using a colorimetric assay kit (BioVision, Milpitas, CA, USA, Catalog #K752-100) following the supplier’s protocol. Serum IL-6 (*N* < 50 pg/mL) levels were measured using a mouse ELISA kit (4A Biotech, Beijing, China, CME0006). Triglyceride levels (1.1–1.8 nmol/µL) were determined using an Abcam (Cambridge, UK) fluorometric kit (AB65336), following the manufacturer’s instructions after liver grinding.

### 2.3. Histological Examination: Evaluation of Steatosis Induction and Lipid Overloading—HE and Oil Red O Staining

#### Tissue Preparation

The liver samples from the mice fed the standard diet (control) or the MCD diet for 2 or 4 weeks were divided in two parts. One part was fixed in 10% neutral-buffered formalin overnight and placed in a paraffin-embedding station (Tissue-Tek VIP5 Jr, Sakura, Villeneuve d’Ascq, France). The paraffinized liver tissue samples were then cut into 4 µm slices using a microtome and stained with a conventional hematoxylin and eosin stain (DRS 2000, Sakura, France). The prepared slides were examined under a light microscope (Nikon, Minato City, Japan, Eclipse Ni-E, 10× objectives). A hepato-pathologist analyzed the blinded slides and scored the samples for steatosis, fibrosis, ballooning, and the NAS score [18].

The other part of the liver tissue was frozen at −80 °C prior to being cut into 9 μm slices, and then stained for lipids using an Oil Red O kit following the manufacturer’s instructions (Biognost, Zagreb, Croatia, ORO-100T). The lipid level was determined via the quantification of the red-stained area (µm^2^)/the area of the field (µm^2^).

### 2.4. Morphological and Metabolic Imaging

#### Methoxyisobutylisonitrile (MIBI) Liver Perfusion

^99m^Tc-MIBI SPECT/CT biodistribution in mice

A total of 20 animals were injected in the retro-orbital venous sinus with ^99m^Tc-MIBI (4.45 ± 0.32 MBq/70 µL), and small-animal SPECT images were continuously acquired immediately after and for up to 15 min post-injection. The quantified SPECT signal was presented as the mean ± SD percentage of the decay-corrected injected dose (%ID). The acquisition of small-animal dynamic planar SPECT was performed using a NanoScan SPECT/CT camera (Mediso, Budapest, Hungary) under ketamine (100 mg/kg) and xylazine (10 mg/kg) anesthesia. A quantitative region-of-interest (ROI) analysis of the small-animal SPECT images was manually performed on the attenuation- and decay-corrected SPECT images using VivoQuant software (v.3.5, InVicro, Boston, MA, USA).

Based on the radiotracer uptake curves, the hepatic uptake was assessed by calculating the mean of the plateau values of the curves (from 120 s after injection to the end of acquisition) divided by the highest value obtained in the first 30 s after injection.

### 2.5. Coherent Anti-Stokes Raman Scattering (CARS) Microscopy

For the CARS microscopy, unfixed liver tissue was used, PBS was added, and a coverslip was placed on top. The images were acquired with CARS microscopy as previously described [19].

An upright microscope (Leica TCS MP5, Wetzlar, Germany) equipped with a Ti:Sapphire laser (Chameleon Ultra II, Coherent, Santa Clara, CA, USA) and an optical parametric oscillator (Chameleon Compact OPO, APE, Berlin, Germany) was used for the CARS imaging. Briefly, the Ti:Sapphire (828 nm) and the OPO signal (1083 nm) outputs were used as the pump and Stokes beams, respectively, to produce a frequency difference of ~2845 cm^−1^. The temporally synchronized laser sources were focused with a 25× water immersion objective (Leica, Wetzlar, Germany, HCX IR APO L 25X 0.95) placed into the sample with a combined power of 30 mW. The same laser sources were used for the simultaneous CARS imaging, two-photon autofluorescence (2P) imagining, and second-harmonic generation (SHG) imaging. The epi-reflected signals were directed into a non-descanned detection unit and spectrally separated with dichroic mirrors and bandpass filters before reaching the photomultiplier tubes (PMTs). The bandpass filters for the SHG, 2P autofluorescence, and CARS signals were 420/40 nm, 535/30 nm, and 700/75 nm, respectively. A 15 µm Z-stack of liver tissues was obtained by taking 2D images of 295 µm × 295 µm along the vertical axis at 5-micron intervals. Both sides of each liver were imaged. The CARS analyses were performed using Fiji software, 2.15.1.

### 2.6. MRI Protocol and Liver Steatosis Quantification

The magnetic resonance (MR) examinations were performed on a Bruker Biospec Avance small-animal MR system equipped with a 4.7 Tesla magnet (Bruker, Ettlingen, Germany), and using a proton volume resonator (diameter of 60 mm, homogeneous length of 80 mm) and an actively decoupled 15 mm surface receive coil (Rapid Biomedical, Rimpar, Germany). The animals were positioned prone on the surface coil, which was mounted on a bed suitable for MRI and with a heating blanket to maintain their body temperature at 37 °C. Before the experiments, the mice were sedated in an induction chamber by the inhalation of a mix of 3% isoflurane with a 2 L/min air flow. During the MR examination, the inhalation anesthesia was maintained with 1–2% of isoflurane in an air flow of 0.6 L/min using a dedicated vaporizer (Ohmeda/General Electric, Milwaukee, WI, USA) to obtain regular breathing frequencies in the range of 90–100 breaths per minute. A balloon was placed on the mouse’s abdomen to control breathing during the examination. A radiofrequency antenna was placed on the mouse’s abdomen, and the animal was inserted into the center of the magnet. A multi-slice T1-weighted image stack was acquired with a spin-echo sequence as the geometrical reference for the subsequent spectroscopy sequences. Liver steatosis was measured by proton magnetic resonance spectroscopy (point-resolved spectroscopy (PRESS) sequence) by positioning two voxels in the liver. The triglyceride (TG) and water peak areas were measured by time-domain fitting (AMARES fitting routines from the MRUI package http://www.mrui.uab.es). Liver steatosis was quantified from the relaxation-corrected ratio of the triglyceride and water signals. The concentrations found in the two voxels were averaged.

### 2.7. Near-Infrared Spectroscopy

#### Data Acquisition

**Figure A1:**
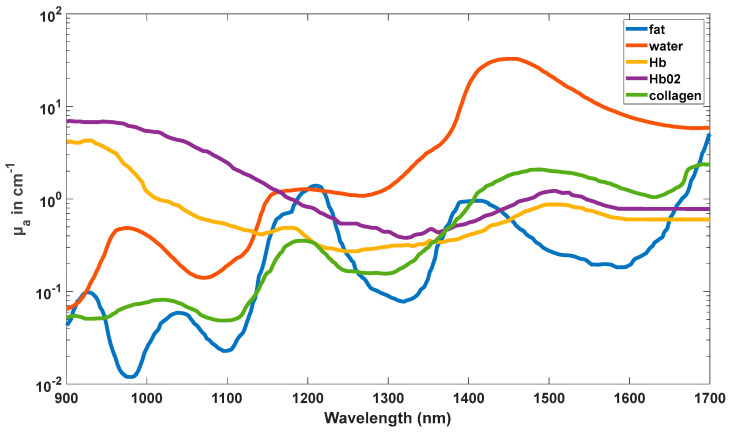
The absorption coefficients of water, lipids, hemoglobin, Hb02, and collagen vs. wavelength from 900 to 1700 nm obtained from the literature. These absorbers correspond to the main ones absorbing near-infrared light in the liver [31].

The NIRS data were acquired using an NIR-SG1 device (InnoSpectra, Hsinchu, Taiwan). All the different spectra were acquired within a 900–1700 nm wavelength range, particularly suitable for the assessment of steatosis, as fat demonstrates a significant absorption peak around 1200 nm, which can be observed on the diffuse reflectance spectra. Each measurement consisted of a repetition of six scans performed for around 10 s in total. Before the measurements of the tissues, the DRS spectrum of a white standard diffuse reflectance sample was acquired (model SRT-99-050, LabSphere, North Sutton, NH, USA) for calibration purposes. After calibrating the model using the fat in the water emulsions, this method was applied to the murine model of liver steatosis using a methionine–choline-deficient (MCD) diet (Figure A1).

### 2.8. Statistical Analysis

The data were analyzed using Graphpad Prism version 10.0 software (GraphPad Software, Inc., La Jolla, CA, USA). The quantitative values were expressed as the mean ± standard error of the mean or median (interquartile range), according to the statistical distribution. The differences between the two groups were compared using a paired samples *t*-test. The differences between the groups fed different diets (control and MCD) were compared using a two-way analysis of variance. *p* < 0.05 was considered to indicate a statistically significant difference. The study of the relationships between the quantitative variables was performed using correlation coefficients (Spearman, according to the statistical distribution). An ROC curve analysis was performed to determine the most discriminative approach in relation to the histological results. Furthermore, linear regressions were conducted to estimate the conversion coefficients between each liver steatosis percentage obtained with one diagnostic tool and the histological percentage obtained by an anatomopathologist’s analysis. The comparison between the independent groups was performed using the following statistical tests: for the quantitative variables, an ANOVA or a Kruskal–Wallis test, as appropriate, and for the categorical data, chi-square tests or Fisher’s exact tests were used.

## 3. Results

### 3.1. Experimental Study Design and Characterization of the Liver Steatosis Model

**Figure 2:**
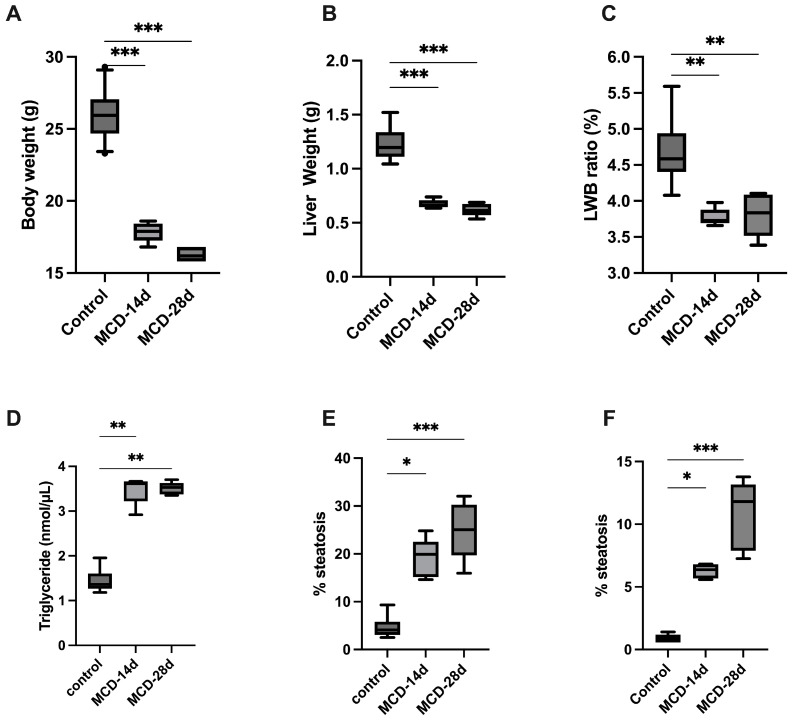
Effect of MCD diet on mice and liver steatosis quantification. C57BL6J mice were fed with normal diet (control), MCD diet for 14 days (MCD-14d), or MCD diet for 28 days (MCD-28d). (**A**) Body weight (g); (**B**) liver weight (g); (**C**) liver-to-body weight (LBW) ratio (%); (**D**) liver triglycerides level (nmol/µL); (**E**) liver steatosis quantification by MRS (magnetic resonance spectroscopy) (%); and (**F**) liver steatosis quantification by CARS microscopy (%). Control vs. MCD, * *p* < 0.05, ** *p* < 0.01, and *** *p* < 0.001.

In the experimental group, the mice were fed a methionine–choline-deficient diet (MCD) for 14 days (MCD-14d) or 28 days (MCD-28d) to induce liver steatosis (Figure 1). The MCD diet led to weight loss due to hypermetabolism caused by an increased sympathetic nervous system outflow to adipose tissue, leading to a less-efficient energy extraction from the nutrients. The mean weight loss was 23.5 ± 0.3% in the MCD-14d group and 27.3 ± 0.9% in the MCD-28d group (*p* = 0.006). In those fed the MCD diet, the weight loss was more pronounced during the first two weeks (Figure 2A). The MCD diet also induced weight loss in the liver (Figure 2B). The LBW was lower in the MCD-fed animals than in the control group (*p* = 0.001) (Figure 2C).

### 3.2. Biochemical Analysis

The serum liver enzyme levels were higher in the experimental group vs. the control group (Table 1), and IL-6 was higher in the mice fed with the MCD diet (79.2 ± 2.3 in the MCD-14d and 251.4 ± 3.1 in the MCD-28d, *p* = 0.01) vs. the control group (46.4 ± 1.3, *p* = 0.006). The level of triglycerides in the liver was higher in the experimental groups (3.1 ± 0.2 nmol/mL in the MCD-14d group and 3.6 ± 0.2 in the MCD-28d group vs. 1.6 ± 0.2 in the control group, *p* = 0.003) (Figure 2D).

### 3.3. Magnetic Resonance Spectroscopy Quantification of Liver Steatosis

The mean percentage of LS quantified with MRS was 7.1 ± 0.8% in the control group, 19.1 ± 1.8% in the MCD-14d group, and 25.1 ± 2.7 in the MCD-28d group (*p* = 0.001) (Figure 2E).

### 3.4. CARS Microscopy

**Figure 3:**
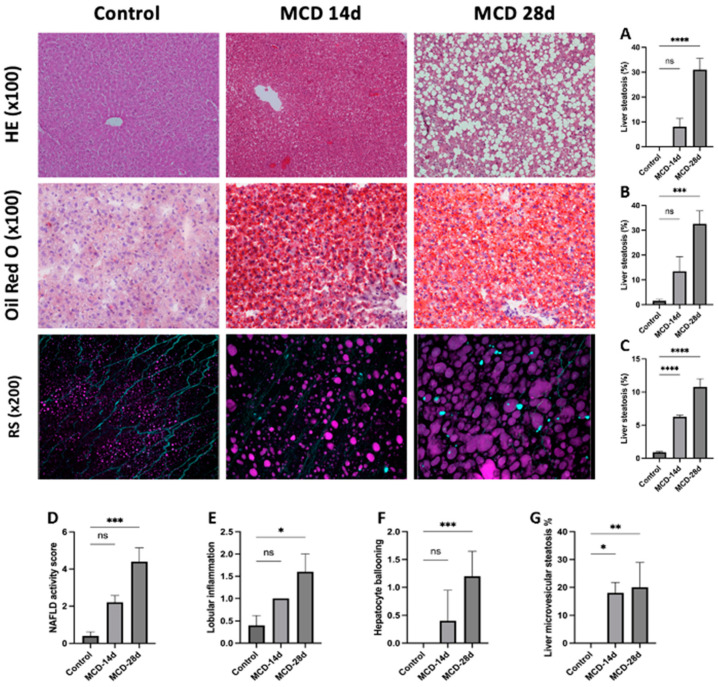
Histological staining (HE ×100), Oil Red O staining, and CARS microscopy (RS ×200) of control, MCD-14d, and MCD-28d groups. Oil Red O staining: lipid droplets are shown in red. RS (×200): lipid droplets are shown in purple. (**A**) HE staining, (**B**) Oil Red O staining, (**C**) CARS microscopy in magenta and SHG and two-photon autofluorescence in cyan. (**D**–**G**) Histological blind quantification by anatomopathologist. (**D**) NAS score, (**E**) lobular inflammation (0–2), (**F**) hepatocyte ballooning (0–3), and (**G**) liver microvesicular steatosis. Control vs. MCD, * *p* < 0.05, ** *p* < 0.01, *** *p* < 0.001, and **** *p* < 0.0001.

CARS microscopy is able to detect liver steatosis by measuring the average lipid density in a sample and by measuring the lipid droplet size (µm) to differentiate between macroV steatosis and microV steatosis (Figure 2F and Figure 3). The average droplet size was 6.3 ± 0.2 in the MCD-14d group (corresponding to microvesicular steatosis) vs. 10.8 ± 1.2 in the MCD-28d group (corresponding to macrovesicular steatosis), with *p* = 0.539 (Table 1).

### 3.5. Histological Analysis

The blind pathologist analysis of the HE-stained liver sections from the MCD-14d group showed macrovesicular steatosis (MV), with large fat droplets displacing the nucleus towards the periphery of the cell. This increased significantly in the MCD-28d group (Figure 3A). The blind quantification showed mild MV steatosis in the MCD-14d group (8 ± 3.3%) vs. moderate MV steatosis in the MCD-28d group (31 ± 4.5%), with *p* = 0.001. The results are summarized in Table 1 (Figure 3). Oil Red O staining quantification based on a semi-automated computerized threshold detection showed similar results: mild MV steatosis in the MCD-14d group (11 ± 2.6%) and moderate steatosis in the MCD-28d group (33 ± 5.2%), with *p* = 0.001. In the experimental group, the level of microvesicular steatosis was similar between the MCD-14d and MCD-28d groups (18 ± 3.7 vs. 20 ± 8.9), with *p* = 0.506 (Figure 3).

Ballooning hepatocytes and lobular inflammation were analyzed to calculate the NAS score [20] (Figure 3). In the MCD-14d group, lobular inflammation and hepatocyte ballooning were rare, and the median NAS score was 2 (1–3); in the MCD-28d group, lobular inflammation and hepatocyte ballooning were higher, and the median NAS score was 4 (3–6), with *p* = 0.560, corresponding to the development of steatohepatitis with a longer duration on the MCD diet. No animals displayed fibrosis in the MCD groups.

### 3.6. ^99m^Tc-MIBI SPECT

**Figure 4:**
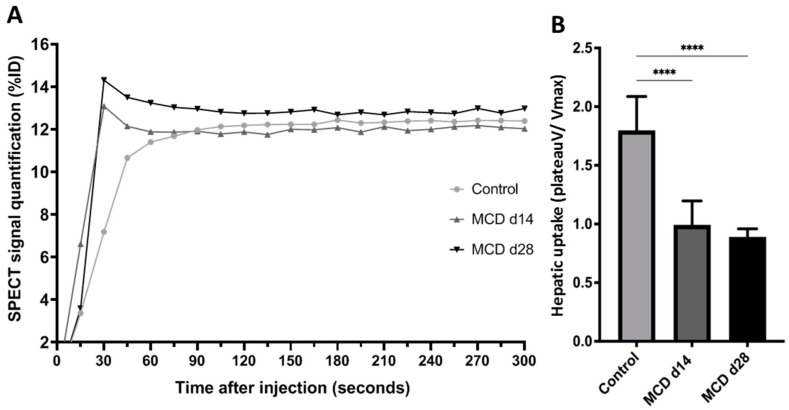
Liver capture via ^99m^TC MIBI scintigraphy. (**A**) ^99m^Tc-MIBI SPECT biodistribution during 300 s after injection. Quantified SPECT signal in liver is presented as mean percentage of decay-corrected injected dose. (**B**) Early hepatic uptake was assessed by averaging plateau values of curves (from 120 s after injection to end of acquisition) divided by highest value obtained in first 30 s after injection. Control vs. MCD, **** *p* < 0.0001.

**Figure 5:**
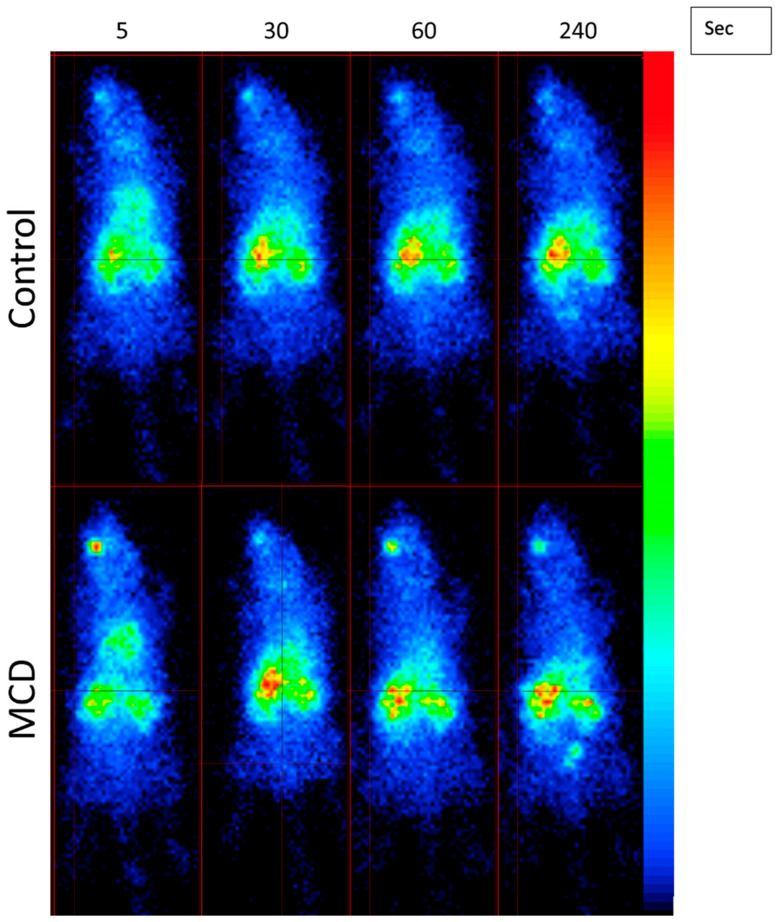
Dynamic SPECT images after injection of 99mTC-MIBI in control and MCD-fed mice at 5, 30, 60, and 240 s. Radiotracer (99mTC-MIBI) was administered retro-orbitally and then captured by liver. The colors are linked to radiotracer capture intensity.

The kinetic curves of radiotracer uptake in the liver differed according to the diet of the mice (Figure 4 and Figure 5). The hepatic radiotracer uptake in the control group increased progressively to reach a maximum value around 120 s after injection, and then remained at a plateau until the end of the acquisition. In the MCD diet groups, the radiotracer levels peaked 45 s after injection before declining to a plateau value, as in the control group. A significant decrease in the mean plateau was observed in the MCD-14d and MCF-28d groups. The mean ratio of the plateau values to Vmax for the first 45 s post-injection decreased significantly in the MCD groups compared to the control group (*p* = 0.009). The ratio was 0.92 (0.9–1.1) in the MCD-14d group and 0.88 (0.8–0.9) in the MCD-28d group vs. 1.86 (1.5–2.1) in the control group, with *p* = 0.009. However, the evolution of the uptake between the MCD-28d and MCD-14d groups was not different (*p* = 0.451).

### 3.7. NIR-SG1

**Figure A2:**
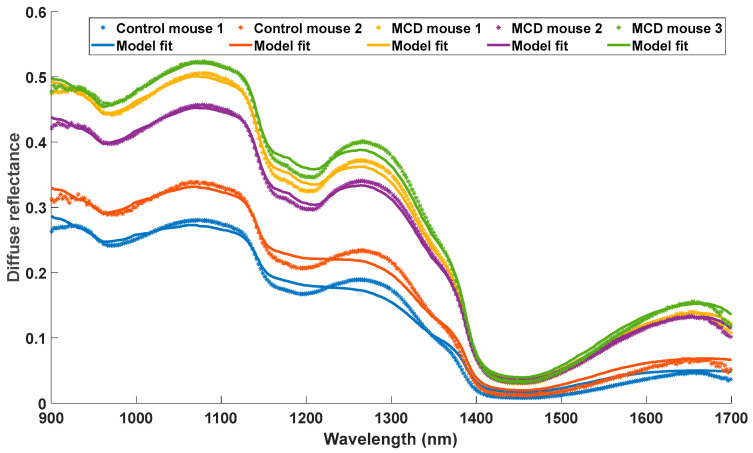
Experimental diffuse reflectance (in arbitrary units) from 900 to 1700 nm of NIR-SG1 acquisition in liver of mice fed with MCD diet or normal diet and corresponding fit obtained using equation. Stars and line plots correspond, respectively, to experimental data and fit obtained. Blue and red plots correspond to control mice, whereas green, yellow, and purple correspond to MCD-fed mice [32].

For the NIR-SG1 acquisitions, mice in the control group or in the MCD-28d group were used. The acquisition was not performed on the MCD-14d group. After calibration, the inverse model with the calibrated parameters was applied and measurements were taken for the mice fed with the normal or MCD diet. The experimental diffuse reflectance spectra and the corresponding fitted spectra are shown in Figure A2 for one measurement taken on each mouse. We can observe a decrease in intensity around 1200 nm, which is due to the high absorption of light. This is related to the absorption peak of fat, which is around 1200 nm. This peak is clearly visible for the MCD-28d mice and is smaller in the control ones, confirming the presence of a high fat content in the MD-28d mice and a lower fat content in control mice. Furthermore, a difference in the overall intensity of the spectra can be clearly seen between the control and MCD-28d mice. This may be due to the high scattering of light by the fat droplets, which then induced an increase in the quantity of light measured. The extracted fat fraction obtained by the inverse model can be compared between the control mice and the MCD-28d mice.

A significant difference between the MCD-28d mice and control mice was obtained: 30.1 ± 0.9 vs. 4.6 ± 1.2, respectively (*p* = 0.001).

### 3.8. Correlation and ROC Curves

**Figure 6:**
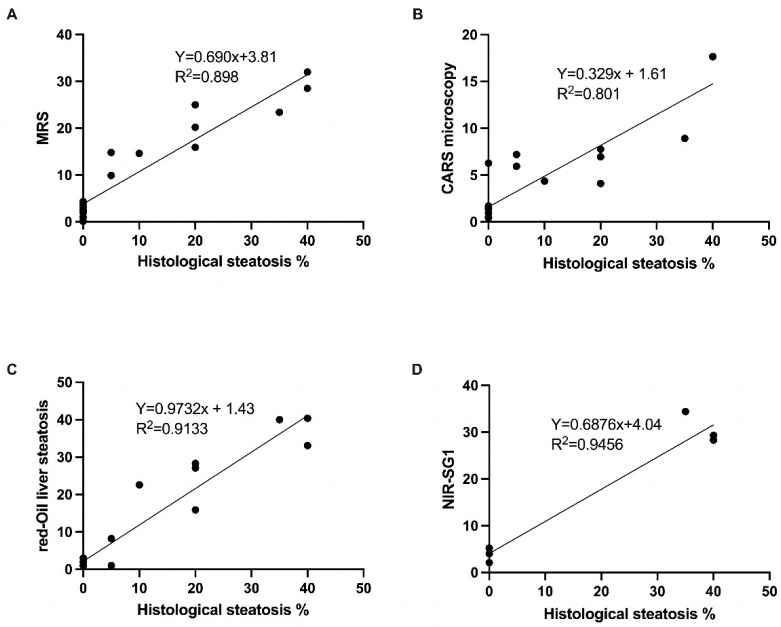
Correlation curves between histological quantifications and each technique. MRS = magnetic resonance spectroscopy; NIR-SG1 = near-infrared spectroscopy. (**A**) Correlation between MRS and histological quantification; (**B**) correlation between CARS microscopy and histological quantification; (**C**) correlation between Oil Red O staining and histological quantification; and (**D**) correlation between NIR-SG1 and histological quantification.

**Figure 7:**
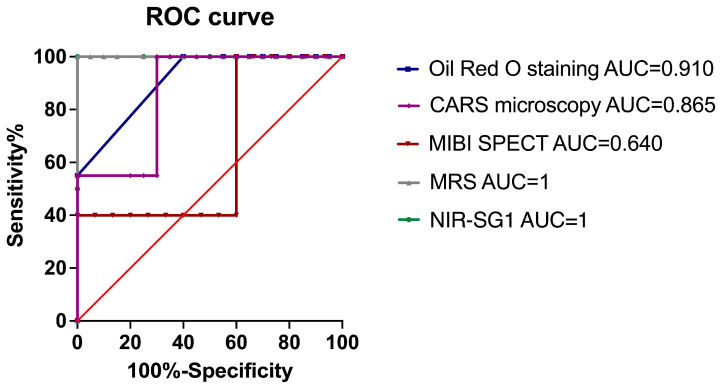
ROC curve for each diagnostic tool versus histological quantification. The red line corresponds to an AUC = 0.5.

The liver steatosis levels based on an NIR-SG1 quantification are highly correlated with those from the blinded pathologist analysis (R^2^ = 0.945) (*p* = 0.001) (Figure 6). A high correlation was found between the CARS microscopy (R^2^ = 0.801) (*p* < 0.001), MRS (R^2^ = 0.898) (*p* < 0.001), and blind pathologist analysis. The ROC curves are shown in Figure 7. The area under the curve obtained was 1 for the NIR-SG1 and MRS (*p* = 0.021 and *p* < 0.001, respectively). The AUC = 0.910 for the Oil Red O stain (*p* < 0.001), and the AUC = 0.865 for the CARS microscopy (*p* < 0.001). The AUC for the ^99m^Tc MIBI SPECT was 0.640 (*p* = 0.013), indicating that it was a less discriminating technique for LS quantification.

## 4. Discussion

This study aimed to compare five diagnostic methods to a histological analysis using a murine model of the MCD diet. The MCD diet model of liver steatosis offers the advantage of providing high reproducibility among animals, leading to a rapid and efficient model of NAFLD with moderate steatosis (≥30%) after 28 days of the diet [21]. The MCD diet model of NAFLD used reproduces the range of histological steatosis, extending from normal tissue to microvesicular and macrovesicular steatosis, alone or in combination. Indeed, two diet duration periods were compared; 14 days of the diet led to mild steatosis (5–29%) with predominantly microvesicular steatosis, while 28 days of the diet led to moderate steatosis (≥30%), corresponding to the clinically relevant thresholds of post-transplantation risk of dysfunction [2,22]. In this study, Oil Red O staining was highly correlated with the histological analysis (R^2^ = 0.913), and was discriminant enough to quantify liver steatosis ≥ 30% (AUC = 0.910). It offers advantages, such as more precise quantification and the identification of mV and MV, avoiding confusion between hepatocyte ballooning and fat droplets, but this technique requires a long and specific sample preparation before quantification [23].

In this study, we showed the excellent correlation between magnetic resonance spectroscopy, CARS microscopy, and near-infrared spectroscopy (NIR-SG1), on the one hand, and the histological quantification of liver steatosis.

MRS is a solid technique for lipid quantification but is less frequently available, especially in cases of organ procurement. It offers the advantage of quantifying the liver steatosis directly for the ROI positioned during the acquisition. In this study, we showed that MRS was highly correlated with the histological quantification (R^2^ = 898) (*p* < 0.001) in a murine model of liver steatosis and precisely determined the steatosis quantification. Furthermore, MRS was able to determine steatosis of more than 30%. In a comparative study of four MR techniques for liver biopsy on patients with MASLD, Boudinaud et al. [24] showed very good correlations between MRS and the histological standard (r = 0.812) (*p* < 0.001).

CARS microscopy allows for the specific imaging of fat droplets in untreated liver tissue for the biological assessment of the fat content in tissue samples. The advantages of CARS microscopy are that no sample preparation is required, and it can precisely and quickly quantify the liver fat content. An interesting aspect of CARS microscopy is the precise measurement of the size of the lipid droplets. In this study, CARS microscopy demonstrated a strong correlation with the liver steatosis quantification (R^2^ = 0.801) (*p* < 0.001), and it was able to differentiate between mV steatosis and Mv steatosis, with an average droplet size of 10.8 ± 1.2 that was only present in the mice fed with an MCD diet for 28 days. The content of hepatic fat was first shown to be strongly correlated (R^2^ = 0.89) with biochemical analysis results by Wu et al. [25] using CARS microscopy. CARS microscopy avoids the laborious procedures required by traditional histopathological examination or biochemical analysis, but it remains a technique that requires a specific and costly microscope system; therefore, miniaturization and portability remain challenging issues for its clinical application [26].

The 99mTc MIBI SPECT/CT imaging technique is used to evaluate hepatic mitochondrial function, which is impaired in liver diseases such as NAFLD. In this study, we showed a significant decrease in the retention rate in the mice fed with an MCD diet vs. the control mice (*p* = 0.009). The rapid washout of 99mTc MIBI, regarding the significant difference between the kinetic curves in the MCD-fed groups and the control groups, confirmed the mitochondrial liver dysfunction the MCD-fed groups. Despite this, the 99mTc MIBI evaluation failed to identify a difference between the two MCD-fed groups, but 99mTc MIBI remains an interesting method, as it evaluates liver function and not only steatosis quantification. Our results are in concordance with those of a study by Rokugawa et al. [9] that showed a decrease in hepatic 99mTc MIBI retention in a rat model of the MCD diet of up to 4 weeks, confirming the correlation between steatosis-related mitochondrial damage and reduced hepatic radiotracer retention.

Near-infrared spectroscopy, using theNIR-SG1, is the most innovative method and the most appropriate device for clinical practice, especially in the case of organ procurement. The device is miniaturized and connected to a smartphone with a Bluetooth application to launch the acquisition. The acquisition takes a few seconds, and the results are available instantly. The calibration of the model has already been carried out using intra-lipid phantoms for grading the fat content. The NIR-SG1 showed a high correlation with the histological quantification, with R^2^ = 0.9456 (*p* = 0.0011) and the AUC = 1 (*p* = 0.021), but the MCD-14d mice were not included. The aim of using the NIR-SG1 was to evaluate this technique in a model with 30% liver steatosis for direct clinical application. Golse et al. [16] recently described a new micro-spectrometer for the quantification of liver steatosis using infrared measurements. This is a very interesting concept study, which included 35 liver measurements in the training cohort and 154 grafts measurement in the validation cohort to detect ≥30% macrovesicular steatosis and achieved a good correlation (R^2^ = 0.81). However, we detected some biases in the application of this technique. First, the method used by Golse et al. to assess liver steatosis involved an algorithm based on a cohort of patients for calibration and a second cohort for validation. In our study, the NIR-SG1 had already been calibrated; thus, each measurement could be used to quantify steatosis. Second, the method used by Golse et al. only quantified macrosteatosis and not microsteatosis or even global steatosis. In our study, global steatosis was quantified.

This study has limitations related to use of the animal model of liver steatosis. Indeed, this model is not exactly correlated to human liver steatosis-related disease, as many other toxins can interact with the liver, e.g., in cases of alcohol consumption and viral hepatitis. Another limit of this study is the quantification of fibrosis with steatosis, lobular inflammation, and the ballooning of hepatocytes. We described a murine model of NAFLD which did not present with liver fibrosis in order to reflect the clinical application of liver grafts more closely, which often have minor fibrosis (≤F2), but the further development of a non-invasive fibrosis diagnosis tool is critical for the histopathological evaluation of MASH. Finally, another limitation of this study is the use of an animal model that, although it can mimic the pathology found in humans, must be interpreted with caution.

The quantification of the steatosis of liver grafts is still an unsolved issue [27,28], and new techniques and devices are needed to assess LS in clinical practice. This is the first study that compares five diagnostic methods to a histological analysis for the quantification of liver steatosis. These methods were Oil Red O staining, MRS, CARS microscopy, 99mTc MIBI SPECT/CT imaging, and a new diagnostic tool, the NIR-SG1 spectrometer from InnoSpectra. Each method possesses advantages and drawbacks when evaluating liver steatosis. We compared several methods for the diagnosis of LS. MRS and CARS microscopy are very effective and precise, as we showed in this study, but are rarely available, especially in the case of organ procurement. US was not considered in this study because it possesses less sensitivity and specificity than an MRI, even though it is much easier to perform. CT scanning (especially dual-energy CT) is an interesting diagnostic technique for LS quantification and is more readily available for organ procurement; however, it was not available for the mice in our center. Dual-energy CT has some drawbacks, and in our study, a dual-energy CT did not improve the performance of CT for LS quantification, especially for mild steatosis quantification [29,30]. The imaging methods that performed best were MRS, CARS microscopy, and the NIR-SG1. Our study paves the way for the use of these promising techniques in a clinical setting. Particularly, the NIR-SG1 should be evaluated in a large clinical study on liver grafts to determine its clinical application.

## Figures and Tables

**Table 1:** Parameters of mice fed with normal diet (Control) and MCD diet for 14 days (MCD-14d) and 28 days (MCD-28d).

Parameter	Control	MCD-14d	MCD-28d
Body weight, g ^£^	25.9 (24.7–27.0)	17.9 (17.2–18.4) ***	16.2 (15.8–16.8) ***
Liver weight, g ^£^	1.2 (1.1–1.3)	0.7 (0.6–0.8) ***	0.6 (0.5–0.7) ***
LBW ratio, % ^£^	4.6 (4.4–4.9)	3.7 (3.6–3.9) *	3.8 (3.5–4.1) **
ALT UI/L ^$^	50.6 ± 8	152 ± 6 *	273 ± 21 **
IL-6 (pg/mL) ^$^	46.4 ± 1	79.2 ± 2 *	251.4 ± 3 **
TG (nmol/µL) (*N* = 1.1–1.8) ^$^	1.6 ± 0.2	3.1 ± 0.2 *	3.6 ± 0.2 **
^(99m)^Tc MIBI Plateau value–max value ratio ^£^	1.86 (1.5–2.1)	0.92 (0.9–1.1) *	0.88 (0.8–0.9) **
MRS (%) ^$^	7.1 ± 0.8	19.1 ± 1.8 **	25.1 ± 2.7 ***
CARS microscopy (%) ^$^ Mean droplet size ^$^	0.74 ± 0.3 0.9 ± 0.1	5.56 ± 0.47 ** 6.3 ± 0.2 **	11.8 ± 1.6 *** 10.8 ± 1.2 ***
NIR-SG1 (%)	4.6 ± 1.2		30.1 ± 0.9 ***
Histological steatosis (%) ^£^ Oil Red O stain	1.4 (1.1–1.9)	11.3 (8.6–19.2) **	33.1 (27.7–37.2) ***
Histological steatosis (%) ^£^	0	8 ± 3.4 **	31 ± 4.6 ***
Moderate liver steatosis (>30%), *n* (%)	0	1 (20) ***	4 (80) ***
Score NAS ^£^	0 (0–1)	2 (1.5–3) **	4 (3–6) ***

Control vs. MCD, * *p* < 0.05, ** *p* < 0.01, and *** *p* < 0.001. LBW = ratio of liver-to-body weight; ALT = alanine aminotransferase (ALT) activity; TG = triglycerides level; ^(99m)^Tc MIBI = ^99m^ technetium methoxyisobutylisonitrile (MIBI); MRS = magnetic resonance spectroscopy; CARS microscopy = coherent anti-Stokes Raman scattering microscopy; NIR-SG1 = near-infrared spectroscopy; NAS = NAFLD activity score. ^$^ mean and standard deviation of mean; ^£^ median (interquartiles).

## Data Availability

The research data are available by request to the corresponding author.

## Abbreviations

ALT	alanine aminotransferase
FLD	fatty liver disease
LS	liver steatosis
LT	liver transplantation
MCD	methionine choline deficient
NAFLD	non-alcoholic fatty liver disease
NASH	non-alcoholic steatohepatitis
MASH	metabolic-associated steatohepatitis
CARS	coherent anti-Stokes Raman scattering
MASLD	metabolic dysfunction-associated steatotic liver disease
mV	microvesicular
MV	macrovesicular
MRI	magnetic resonance imaging
MRS	magnetic resonance spectroscopy
^99m^Tc MIBI	technetium-99m-2-methoxyisobutyl-isonitrile
ORO	Oil Red O staining
RS	Raman spectroscopy
NIRS	near-infrared spectroscopy
LBW	liver-to-body weight ratio
AUC	area under the curve
